# Experiences of nurses educated outside the European Union of a Swedish bridging program and the program’s role in their integration into the nursing profession: a qualitative interview study

**DOI:** 10.1186/s12912-020-00525-8

**Published:** 2021-01-05

**Authors:** Emina Hadziabdic, Anna-Maria Sarstrand Marekovic, Johanna Salomonsson, Kristiina Heikkilä

**Affiliations:** 1 Department of Health and Caring Sciences, Faculty of Health and Life Sciences, Linnaeus University, SE-351 95 Växjö, Sweden; 2 Department of Social Studies, Faculty of Social Sciences, Linnaeus University, SE-351 95 Växjö, Sweden; 3 Department of Swedish, Faculty of Arts and Humanities, Linnaeus University, SE-351 95 Växjö, Sweden; 4 Department of Health and Caring Sciences, Faculty of Health and Life Sciences, Linnaeus University, 391 82 Kalmar, Sweden

**Keywords:** Bridging program experience, Nurses educated outside the EU/EES, Qualitative interview study, Transition

## Abstract

**Background:**

Countries all over the world are experiencing a shortage of registered nurses (RNs). Therefore, some countries, including Sweden, have tried to solve this by recruiting internationally educated nurses (IENs). Countries offer bridging programs as educational support to qualify IENs for nursing work in the destination country. However, there is little research on IENs’ experiences of bridging programs in European countries and how these programs facilitate their integration into the world of work and their new society. The aim of this study is to explore the experiences of nurses, originally educated outside the EU (European Union)/EES, of the Swedish bridging program and of the program’s role in facilitating their integration into the nursing profession in Sweden.

**Methods:**

A qualitative descriptive design was used to explore the topic based on 11 informants’ perspectives and experiences. Purposive sampling was used to recruit participants at one university in Sweden. Data were collected by individual interviews using a semi- structured interview guide during the year 2019 and were analysed using an interpretative thematic approach.

**Results:**

Two main themes emerged from the analysis: 1) Return to nursing, and 2) The bridging program as a tool for transition to nursing in Sweden. The first theme includes conditions and experiences such as personal motivation and determination, and support from others that the participants described as important in order to achieve the goal of re-establishing themselves as registered nurses in Sweden. Furthermore, the second theme describes the participants’ experiences of the bridging program as mostly positive because it led to new learning and achievements that were valuable for the transition to nursing in Sweden; however, the participants also emphasised the challenges of their transition into the nursing profession, which were related to instances of misrecognition of their professional competence and the uncertain outcome of the program.

**Conclusions:**

This study found that the bridging program facilitated integration into the nursing profession for nurses educated outside the EU/EES, especially knowledge gained in clinical-based training. Thus, it is important to recognise and value the IENs’ experience and previous knowledge and training when developing the bridging program’s curriculum.

**Supplementary Information:**

The online version contains supplementary material available at 10.1186/s12912-020-00525-8.

## Background

The global shortage of registered nurses (RNs) has been shown to have negative consequences on healthcare, such as increased mortality, staff violence, accidents, cross-infection and adverse postoperative events. Therefore, some countries have tried to remedy shortfalls in the nursing workforce via the recruitment (including spontaneous migration) of internationally educated nurses (IENs) [1–3].

From an international perspective, registered nurses differ in terms of knowledge base, regulations regarding what nurses are allowed to do, as well as differences in nursing education, national health care systems, nursing practice and cultural values. The bridging program is aimed at internationally educated nurses with a degree from a country outside the EU (European Union)/EES. After completing the bridging program, the IEN must show the knowledge and ability required to be able to apply for nursing license in Sweden [4]. Consequently, it is necessary to offer bridging programs to IENs to ease their transition and expand their cultural, practical and theoretical knowledge to ensure patient safety [5, 6].

There are literature reviews about IENs’ experiences related to migration and acculturation [7], migration and transition into the Canadian health care system [8, 9], bridging programs [6] and work experiences in a new country [4, 10, 11]. Such research shows that IENs experience the migration and transitioning mostly positively in terms of improved income and professional status. However, they also experience cultural displacement which appears largely to stem from communication problems and language differences, the feeling of being an outsider, and differences in nursing practice [9]. It has been shown that to achieve a successful transition, IENs need education and support [5, 12, 13] to expand their understanding of the receiving country’s educational philosophy, gain occupation-specific language training and clinical time [14]. In particular, bridging education programs are crucial to facilitate acculturation and to promote cultural congruence in the healthcare system [15] and are also a way to provide safe and high-quality healthcare and to maintain the responsibility of nursing as a regulated profession [5, 12, 13].

As shown above, the significance of bridging programs for a successful transition to the nursing profession upon migration cannot be ignored. Yet our review revealed that research on IENs’ experiences of bridging programs in European countries and how these programs facilitate integration into the world of work as well as society in general is limited. This is an important area of study because nurses educated outside the EU are an under-used, under-appreciated human resource, and represent a means to remedy the nursing shortage. Knowledge about these programs and the experiences of participants is also vital to facilitate the transition into the labour market with the appropriate skill level [3]. Our study is a contribution to this emergent field of study.

### Bridging program for nurses educated outside the EU/EEES working in Sweden

Bridging education programs for internationally educated nurses have existed in Sweden since the beginning of the 1990s, [16]. The aim of these programs is to transfer and make use of knowledge and skills that have been obtained in countries outside the EU, i.e. the participants are able to supplement their education to qualify for nursing work in Sweden. The program is financed through a special grant from the Swedish government and the level of funding has increased significantly from SEK 51 million in 2012 to SEK 202 million in 2018 [16].

The bridging program is a one-year course and is presently offered at five universities in Sweden in accordance with Swedish law on Higher Education (2008: 1101). The Higher Education Law (2008: 1101) states that education must be planned to consider each student’s previous education and work experience. The bridging program includes different courses containing education about the nursing profession, nursing, pharmacology and pharmaceutical calculation as well as learning and leadership. In addition, the clinical training components are carried out in primary care, municipal care and nursing as well as in one of the specialties of medicine, surgery or geriatrics.

After the bridging program, nurses educated outside the EU have to pass a so-called knowledge test, which is in Swedish and examines areas such as laws and regulations for the health care sector. It also includes a practical test showing the clinical skills nurses need before they can work as registered nurses in Swedish health care. Seventy-nine percent of nurses who completed the bridging program had acquired a good position in the labour market after 1 year [16].

## Aim

The aim of the study is to explore the experiences of nurses, originally educated outside the EU/EEES, of the Swedish bridging program and of the program’s role in their integration into the nursing profession in Sweden.

## Methods

A qualitative, interpretative and descriptive design was used to explore the topic based on the informants’ perspectives and experiences in order to capture a variety of understandings and to gain an in-depth knowledge of the studied phenomenon [17].

### Procedure

Purposive sampling [18] was used to recruit participants who had completed, or were at the end of the bridging program at one university in Sweden in two cohorts. The first five interviews were conducted in the early spring of 2019 with participants who had just completed the program. The following six interviews were conducted in late 2019 with participants who were at the end of the program. The procedures to access the informants were the same. First, ethical vetting was sought and approved by the Swedish Ethical Review Authority. Thereafter, the fourth author (KH) contacted the program coordinator for the bridging program by phone and e-mail in order to obtain permission for the study. After the approval was obtained, the program administrator was contacted to provide contact details of the students. The authors then reached out to all of the students from both cohorts by phone and e-mail and provided verbal and written information about the aim and procedures of the study, along with ethical considerations. The participants who were interested in participating in the study gave their consent, and the time and place for the interview was decided.

A total of 11 bridging program participants were included in the study, in order to ensure deep and rich information and better understanding about the phenomenon of interest [17]. The participants comprised a heterogeneous group in regard to gender (three men and eight women), country of origin (three from Europe, five from Asia and three from Africa), age (mid 20s to mid-50s), reasons for migrating (refugees, family reunification and labour migrants) as well as how long they had lived in Sweden before the interview (4–18 years) (see Table 1).

**Table 1:** Characteristics of the study population

Variable	Persons (*N* = 11)
Female	8
Men	3
Age (years)	
29–39 years	5
40–50 years	5
51–61 years	1
Migrating to Sweden	
Year 2003–2009	3
Year 2012–2018	8
Place of birth	
Ethiopia	2
Gambia	1
Iran	1
Belarus	2
Syria	2
Bulgaria	1
Kyrgyzstan	1
Armenia	1
Country where nursing training was completed	
Ethiopia	2
Gambia	1
Iran	1
Belarus	2
Syria	2
Turkey	1
Russia	1
Armenia	1

### Data collection

Data were collected during 2019 through individual interviews using a semi-structured interview guide to allow for a conversational style of interviewing. The interview guide was developed specifically for this study and included three main areas covering the interviewees’ educational and professional experience before arrival in Sweden; educational experience of the bridging program and clinical training; and expectations of future working life. Each theme was supplemented with 5-12 open-ended questions. Examples of questions that were asked are: “Can you tell us how you came to be a nurse in the first place?”, “Can you tell us how come you applied for the bridging program?”, “Can you tell us about your experience of the bridging program?”, “What was different and what was familiar when working as a nurse compared to previous experience?”, and “How do you envision your near/distant future in regard to working and professional life?”. Furthermore, the researchers tried to stay responsive to the interviewees’ reflections and followed up with questions to expand on their different experiences or to clarify reasoning and details if needed. The interviews were conducted individually or in pairs by female authors EH, A-M SM and JS either face to face in a secluded room at the university or by telephone, depending on what the participant opted for. All authors had extensive experience in conducting qualitative research on migration issues. Eight interviews were conducted face to face in a secluded room at the university, and three by telephone. The interviews were held in Swedish and lasted 45–75 min. All interviews were audiotaped and transcribed verbatim by professional secretaries before analysis. Numbers are used to represent the IENs to ensure anonymity.

### Data analysis

The data was analysed using Ödman’s interpretative approach [19] as the starting point to allow for a comprehensive understanding of the meaning the participants attributed to their experience of the bridging program. The transcripts were read several times by all authors to understand the overall picture of the experiences expressed. The fourth author (KH) developed the first tentative interpretations based on a subset of four interviews. These interpretations were then developed and refined by including the additional seven transcripts by the second author (A-M SM), thus increasing and solidifying the codes. All of the coding was done manually and included color-coding, and writing notes and memos on all transcripts. The second author (A-M SM) further expanded the analysis by refining and reorganizing the preliminary codes into themes and sub-themes until a coherent understanding of the data was achieved. During this phase, alterations were made primarily to reduce the number of sub-themes, e.g., two sub-themes were merged into one. This process allowed for the analysis to reach internal homogeneity and external heterogeneity. The themes were further revised both in relation to the initial codes and by returning to the whole data set in an iterative process, thus following the thematic analytical model of [20]. Finally, the main themes: ‘Return to nursing’ and ‘The bridging program as a tool for transition to nursing in Sweden’ were named along with the sub-themes (see Table 2). All authors reviewed and checked the content and grouping of the themes and validated the interpretations in different phases and thus helped to refine and develop the interpretations to ensure credibility. Data sampling and data analysis proceeded until no new information was acquired. Confirmability of the results was strengthened by supporting the themes and sub-themes with illustrative quotations throughout the results section and by presenting the analysis in a coherent way [18].

**Table 2:** IEN: experiences of the bridging program in Sweden illustrated in two main themes with accompanying sub-themes

Main themes	Sub-themes
1. Return to nursing	1. Motivation and determination
	2. Support from others
2. The bridging program as a tool for transition to nursing in Sweden	1. New learnings
	2. Disappointments, achievements and future plans

## Results

The analysis revealed two main themes: *Return to nursing* and *The bridging program as a tool for transition to nursing in Sweden* (see Table 2). The first theme includes conditions and experiences which the participants interpreted as important for achieving the goal to re-establish themselves as registered nurses in Sweden. Sub-themes are: *Motivation and determination* and *Support from others*. The second theme reveals the participants’ experiences of the bridging program and how the program aided and challenged their integration into the nursing profession and includes the sub-themes; *New learnings* and *Disappointments, achievements and future plans*.

### Return to nursing

The goal to return to the nursing profession was consistently emphasized by all participants in the study; “It has always been my goal since I came to Sweden” (P3) or “My goal was to get the licence [to work as an RN] as soon as possible” (P8). However, the path leading to the start of the program varied in length, from only a couple of years up to almost 15 years. Seven of the participants started the program within a five-year period after arrival in Sweden. The participants described personal as well as social conditions they deemed important for a successful transition to the world of work as a nurse. Motivation and determination in terms of a clear understanding of essential preconditions and how to acquire them were important personal conditions, while support from formal and informal relations were important social conditions.

#### Motivation and determination

The participants presented themselves as persons with a strong will, who wanted to reach their goal to obtain Swedish registration as a nurse, and as persons who worked in a dedicated and purposeful way to achieve the goal. The participants described two basic conditions as important for reaching their goal: acquiring Swedish language competency and work experience from the Swedish health care sector.

Several of the students did not regard the Swedish language as an obstacle in itself for their life and studies. However, they recognized the importance of acquiring language competencies to succeed in the world of work. Therefore, they tried to find ways to acquire the language skills and qualifications that were needed. They had, sometimes very intensively, studied Swedish language from the beginning by starting basic courses in Swedish for immigrants (SFI) and then had continued to meet the requirements for academic level studies. They also developed different strategies to learn Swedish which included e.g., reading books, watching TV and spending time with Swedish-speaking friends.

You just have to keep trying and stay curious [ … ] and study like I do now. I’m not a person who says, ‘Okay I’m finished one with my education’ and close all books. No! I read every day, because I can read this paper today and then tomorrow [when I read it again] I will find new information that I didn’t find yesterday. [ … ] To learn a new language when you’re an adult is not easy, but we learn every day. I spend time with colleagues and Swedish people [to learn]. (P1).

Formal certificates confirming appropriate levels of Swedish were obtained through language courses (SFI), often specifically targeting immigrants with experience of working in healthcare. The courses also included practical language training at different health care establishments. These courses were described as significant by the informants for assisting them in their endeavour to work as an RN in Sweden, as they not only provided formal competence in Swedish language but also gave essential knowledge and insights about the Swedish healthcare system. Moreover, many of the participants also took extra measures to improve their knowledge and the chances of reaching their goal, either by working part-time in health care as assistant nurses and studying at the same time, or by taking complementary courses.

I started a course in basic Swedish [Swedish as a second language, SSL]. After one month, in March, I started to work as an assistant nurse, employed by the hour, and studied at the same time. When I finished basic Swedish, I was employed. I continued to study SSL levels 1, 2 and 3 as distance learning courses while working 85% at a retirement home. (P2)

The participants recognized that it was hard work, working and studying at the same time. But they were well aware that a successful transition demanded experience of working in Swedish health care and learning the Swedish language so they could manage the daily nursing practice. They reported the value of this hard work during the bridging program and subsequent transition to the world of work as it gave them not only language competence but also knowledge of the Swedish healthcare system. One of the participants described the experience she gained in Sweden before starting the bridging program as valuable because it made her aware that “[ …] I needed to develop my Swedish [ …] and I got an initial overview of the [Swedish] society and the differences in nursing compared to [country of origin]. (P3). However, the participants seldom explicitly described themselves as determined, but they often stated that “there is no other way, it just has to work out”.

#### Support from others

The participants described social relationships as significant for their experiences of the bridging program and their chances of a successful return to nursing. These contacts and relationships were both formal and informal in nature.

The participants’ experiences of support from the authorities varied. In some cases, the participants felt the authorities had, hindered them from striving to get the education they needed, or that they needed to take matters into their own hands.

They [officials at the Swedish public employment agency] asked me: ‘How did you manage to get a trainee position without our help?’ There were not pleased and wondered how I had managed to sidestep them. But I said, I’m here and I need your signature. I will not leave before I get the signature because I have found a trainee position. (P3)

However, the participants also spoke about other persons, i.e., their managers or supervisors where they worked, who had encouraged them and informed them about the possibilities to get the Swedish registration. For some of the participants, it had also been possible to receive their education during a leave of absence from their workplace. Some of the participants, primarily newly arrived refugees or asylum-seekers, were also targeted to more general investments for competence by the local authorities, for example ‘fast-track’ courses for healthcare professionals.

It was just a coincidence that I was accepted [to the language training program for newly arrived healthcare professionals]. I was playing football and a friend accidentally hit me over the ear, so I had to go to the district health care center. The nurse at the center knew I was a nurse and told me about the local project and she signed me up for it. [And later] I was accepted for the course. (P4)

These formal relationships and acts of encouragement described by the participants were important drivers in learning about the possibilities to supplement one’s education and to set favourable conditions for the bridging program.

Also, the informal networks and relationships helped the participants to manage the bridging program. Many of the participants had family responsibilities, material as well as emotional, that needed to be managed and fulfilled if they were to go back to school. During the education, several participants received their main support from their family members, especially from spouses or parents who took responsibility for the household tasks and childcare and shared economical expenses, so that the participants could devote all their time to their studies and pursue their goal.

Interviewer: What was it like to attend the bridging program while having small children, what did that demand from you?

Participant: Her [daughter’s] dad is with her all the time. If I need to relax and study, he’s with her. Sometimes, as a mother, I think that he is responsible for her and it is not my problem because I am a student. (P5)

The value of this social support system in bridging program was highlighted in several accounts, especially by those with families and children. The support was important for creating the possibility to devote the necessary time to studies. Support from spouses was also important for sharing the emotional and care responsibilities in the families that are traditionally accepted by mothers, which is illustrated well in the excerpt above. For participants both with and without family responsibilities, the support from fellow students was highlighted. The participants described the strength of being part of a program where they all shared occupational experiences and a common goal, despite their differences in terms of national background, gender, age etc. The differences were even described as a resource:

We all have different skills, experiences and capabilities but it was a really good student group because we worked together and helped each other. Some had worked for many years [as nurses] and others had not [ …] as a whole it was a really good group, we cooperated and assisted each other. [ …] We come from different countries and have different knowledge so of course we are different, but we give information, become friends and study together. (P6)

The participants could make use of each other’s competencies and knowledge to advance in the program and to encourage each other to keep striving.

The support from informal social relationships, such as family and friends and fellow students was emphasized as vital for encouraging and enabling their perseverance in the bridging program. For many participants the decision to enter a bridging program was a huge investment, both financially and emotionally, and the value they put on the support from near ones as a key to their success should not be understated. Formal social contacts and connections, such as public officials and authorities were mostly viewed as significant for learning about the formal requirements for working as an RN in Sweden and becoming familiar with the health care system.

### The bridging program as a tool for transition to nursing in Sweden

All participants considered that taking part in the bridging program was a more reliable option for reaching the goal of working as an RN in Sweden, compared to preparing on their own for the test that precedes nurse registration. The program thus provided a clear path toward their goal and a chance to fulfil the requirements, and to learn about the Swedish healthcare system and the role of RNs. The general perception expressed was positive in terms of the program as a tool for reaching one’s goal, nevertheless, the participants also reflected on obstacles and drawbacks.

#### New learnings

Attending the bridging program contributed more, according to the participants, to learning and understanding the differences in the scope of nursing practice in Sweden compared to their previous experiences. The main difference in nursing practice described by the participants concerned the relation between nurse and patient in daily practice. The autonomy of patients and the patient-centred care in Sweden were seen to differ greatly from what they had been used to.

The difference is that you must consider the whole person, not just the disease. I must always ask the patient before I do anything, get consent if I wish to examine him and ask if he wants the treatment. The patients have a right to decline treatment. That was most important to learn, that I must ask the patient before I do anything. It is different in Syria, where the patient is treated without consent. He is there [at the hospital] and [therefore] accepts treatment. (P7)

Many of the participants were not accustomed to the autonomy of the patients, and the need to confer with them before treating them. This was a new learning to handle, although they experienced it as positive. They recognized it to be important knowledge to acquire and that the bridging program contributed to the acquisition of that knowledge. Generally, the theoretical courses, which related to medical competencies and pharmacology, were seen by many as repetitive or affirmative of the knowledge they had already attained in their original nursing education and working life experience.

I didn’t learn much new about the medical aspects [of nursing] but I learned a lot about the [organisation of the] medical system and routines, how to write in the journals etc., because that is different everywhere. The medical aspects are universal, so I didn’t learn new things because I had already done so. (P10)

The participants regarded the practical courses as having a greater value for their learning and prospects of becoming a Swedish RN. The clinical training, which was part of the bridging program, was particularly appreciated. During clinical training the participants reported learning about the Swedish healthcare system, how healthcare care is organized in different establishments and how the forms of care differ, for example primary care, home care and hospital care. Clinical training also contributed to a deeper understanding of the scope of nursing, e.g., daily routines and assignments, relations to different professional groups that nurses collaborate with in daily practice, and work division between professional groups.

Clinical training is difficult, but it gets easier once you know more about your role, when I know what my job assignments are and what assignments the doctor has. But it takes time, I need more time [in clinical training] to feel confident, or how to put it. [ …] You learn in daily practice. If a group needs support for example, who does that? Is it perhaps the doctor? Or the assistant nurse? Or me? (P4)

The clinical training was described as challenging, but the participants still valued the learning that took place there rather than in classrooms. They had seen how the roles between doctors and nurses differed from their home countries, where the doctors had a more authoritarian role than in Sweden. To successfully adapt to the new role was time-consuming but was provided by the clinical training. The participants’ general stance was that this type of knowledge can only be acquired through experience of practical work and interaction at the different workplaces, as the scope of nursing and also the vocabulary varies between different hospital wards or healthcare establishments.

Those who work in medical wards use their special concepts and those who work in surgery have theirs. That is why I have suggested that the clinical training needs to last at least six months to give us a chance to see different wards. For example, I only spent ten days in home care and one month in primary care. That was not a lot of time [to learn]. (P4)

Clinical training also placed the participants in challenging situations, and they faced difficult assignments. The advanced materials nurses had access to and worked with in Swedish health care, e.g., the digital solutions, such as journal systems, presented a challenge. Some of them regarded this as a problem, as their technological knowledge was not that good. Journaling and documentation were also described as difficult because it challenged the participants’ language competencies.

I would put more focus on documentation. I think most of us have inadequate knowledge, not just me. [ …] When you sit down [to do it] you don’t know which box [to check] or what to write. It is difficult. I see how competent the nurses are. But I panic. It takes time for me and it stresses me out. [ …] Perhaps my colleagues will say ‘you have spelled this wrong or made a mistake here’. (P9)

As illustrated in the excerpt, the participants expressed insecurity about language competencies related to documentation and journaling, which were often seen as new routines, although central to the nurses’ scope of practice. Due to experiences like this the participants valued clinical practice and described it as a means to improve their language competency, technical knowledge and familiarity with work routines, and therefore requested more clinical practice during the bridging program.

#### Disappointments, achievements and future plans

As mentioned, the general experience of the bridging program amongst the participants taking part in this study was positive. The knowledge the participants gained throughout the course of the program, particularly during clinical practice, was identified as vital for a successful transition to the world of work and to re-establish oneself as an RN. Nonetheless, some participants also reported experiences of misunderstandings of their role and competencies during clinical practice. The participants described a lack of knowledge about the bridging program and the participants’ professional backgrounds among some of the supervisors.

Sometimes they [supervisors of clinical practice] do not understand what the bridging program is and the [competency] level we have. I tried to explain to my supervisor that I was not there for language training but for the bridging program and that I was already an educated nurse. But for four weeks she only addressed me as a ‘language learner’ [språkpraktikant]. (P7)

Not getting recognition for one’s professional competence while attending clinical practice due to the supervisor’s lack of knowledge is one example of the disappointing experiences the participants described.

A second example related to the uncertainties of the outcome of the program as it did not guarantee acquisition of a nursing licence from The National Board of Health and Welfare. One participant described how the language competencies of some of the participants were perceived by the supervisors in clinical practice as problematic and as affecting the participant’s ability to work as an RN. One participant said: ‘there were some problems during the clinical practice where they complained about our language competency and said that we should work as assistant nurses instead’ (P2). The same participant continued to question why the risk of not passing the program was delivered late and wished that the program directors had made this clear earlier. Although she did not worry about her own future, she expressed compassion for her fellow students who had not managed to get through the education. They all had invested much time and energy in the education, and she empathized with those for whom it had not “paid off”. For most of the participants though, having managed the education and especially the clinical training was both a relief and an achievement that they were proud of:

I was nervous up until the last day. When the examiner told me that I had passed [the program] I couldn’t believe what I’d heard so I had to ask her again. It was such an amazing feeling! I am proud and very glad that this was possible because it was a long journey, a really hard journey. (P3)

The possibility to work as a registered nurse while waiting for the registration was appreciated and anticipated. However, for 9 of the 11 participants, the bridging program was not the final goal but instead it had made possible new goals for their future and working life. Most of them planned to work as an RN for a few years to gain experience and then continue their education and become a specialist nurse in an area of particular interest. Especially popular was the idea of becoming a district nurse, but other specializations were also of interest:

In due time, I plan to continue my education and become a specialist nurse. [ …] Primarily to specialize as a district nurse, but it depends … perhaps specializing in diabetic care, asthmatic care or something like that. (P8)

My idea is to work at the hospital for a couple of years and then if all goes as planned, I will continue my education. [ …] My main interest is to specialize as a nurse in intensive care. (P6)

## Discussion

Overall, the study found that the participants expressed generally positive experiences of the bridging program and that it had a significant role in aiding their transition into nursing in Sweden, which is a finding consistent with previous research [5, 6, 14].

The analysis highlighted conditions and prerequisites which the participants interpreted as important for achieving the goal of re-establishing themselves as registered nurses in Sweden. The importance of acquiring language proficiency and gaining experience from working in the Swedish health care sector which required personal motivation and determination to cope with various demands, was stated by all participants. This is a finding not previously described in European research on IENs. However, the struggles of integration into nursing for IENs in Canada and the importance of perseverance are discussed by Covell et al. [21] which lends support to our results. This study thus found that awareness and engagement, changing of attitude and the positive meaning of obtaining a nursing license facilitated a healthy transition into the workplace [22].

Furthermore, the participants also described receiving support from family, friends and fellow program participants as well as from public authorities and managers as important for facilitating the transition to nursing in Sweden. The importance of material and emotional support from close social relations such as family and friends was emphasised in all interviews. In this study, the participants continued social relationships from old networks and also developed relationships with new networks such as networks of fellow program students. An interesting finding from this study is that the support from formal organizational resources varied considerably. Most positively described was the support from managers at workplaces who offered encouragement, information on formal requirements for practicing nursing in Sweden and practical help, i.e., temporary training leave, which enabled the participants to attend the program. Participants who had received refugee status were also aided by formal programs targeting newly arrived professionals. However, there were examples where participants had experienced discouragement from public officials or difficulties in navigating and understanding the possibilities and demands of the system.

These results highlight that more support is needed from formal organizational resources, i.e., information about the application process, the requirements for attending bridging programs, financial support available during this process [4] etc. This is particularly the case for individuals migrating for other reasons than being refugees, as these individuals are not targeted by programs for the newly arrived and are thus left to cater for themselves to a greater extent [23]. The centrality of employers in identifying IENs working as assistant nurses and supporting their process of returning to nursing as an RN is also an imperative and new result of this research.

In line with results from existing research our study revealed that attending the bridging program equipped the participants with valuable and practical new learning that facilitated the transition to work as an RN in Sweden [5, 6, 14]. Theoretical courses focusing on the scope of nursing practice in a Swedish context were particularly appreciated as they offered insights into how professional relations and nurse-patient relations differed compared to their previous education and professional experiences in the home country. The patient-centred care was described as a new and unfamiliar approach by the participants. These findings support previous qualitative research that found bridging programs to be valuable because they contribute to knowledge on how nursing is practiced in the destination country [6, 7, 11]. The participants could also draw on knowledge acquired from past education and professional experience, especially in areas that demanded medical and pharmacological competencies [8]. Therefore, understanding nurse’s roles, performance and skills should receive more consideration when tailoring educational program for nurses educated outside the EU/EES [11, 14]. Moreover, recognising and valuing the IENs’ experiences and previous knowledge should also be given priority in the educational programs.

Experiences gained in clinical training were highly valued by the participants, who emphasised the importance of ‘learning-by-doing’ when they were about to make the transition into nursing in Sweden. Areas such as independent decision-making of nurses, teamwork and collaboration were described as skills that could not be taught in class but needed to be experienced first-hand, and the participants suggested they should be included to a greater extent in the bridging program. But clinical practice also brought out challenging experiences of misrecognition of professional competence and misunderstandings and insecurities due to language barriers. Language proficiency affected the performance in the bridging program, clinical practice, and the knowledge test to obtain a nursing license in Sweden. The study found that medical terminology, abbreviations and names of medication and equipment varied between countries and this complicated clinical work. Communication barriers have an impact on healthcare in areas such as patients’ satisfaction with the healthcare [24] and patient safety [25]. It is therefore essential that language skills are taught during the bridging education program.

Our results differ to some degree compared to previous research. In our study the participants did not report being lonely and did not experience discrimination, in contrast to previous studies [4, 7, 8, 10]. One possible explanation could be the differences in the methods used or a bias in the selection of participants to this study. Another explanation could be that differences in the reasons for migrating (refugees, family reunification and labour migrants) affect whether or not people have a healthy transition [22]. The overwhelmingly positive experiences reported by the participants in this study are also revealed in their future projections as all but one participant talked about continuing their education to become a specialist nurse.

### Limitations of the study

A limitation of the study could be the recruitment procedure of the participants in the study. In the process of selecting participants for the study we contacted all program participants from two cohorts with the intention of capturing a variety of experiences. The participants who declined to partake in the study might have felt a threat to their independence, e.g., program participants who did not complete the program may have been afraid to express their views and this may have affected the results of the study [17]. The next step for future studies is to include the perspective from the participants who did not complete the program. However, this research gave a deeper understanding of the phenomenon studied and supported the method in terms of the interview guide and recruitment approach so that findings can be transferred to other similar settings [17].

## Conclusion

This study found that bridging program helped nurses educated outside the EU/EEES to achieve their goal, i.e. to obtain a nursing license in Sweden. However, achieving goals was also dependent on one’s personal characteristics such as strong will and knowledge, professional experience, and language proficiency, as well as development opportunities that were available such as formal and informal support. The findings from this study have implications: 1) for academy staff when developing the bridging programs’ curricula by recognising and valuing the IENs’ experience and previous knowledge and the importance of training and supporting supervisors in IENs clinical-based training, 2) for decision-makers and authorities when developing policies concerning: language programs, the information provided to applicants about the application process, the required bridging program and financial support available during this process to address IENs’ specific conditions and 3) for participants, due to the significance of being aware of the importance of material and emotional support from close social relations such as family and friends during the bridging program.

## Supplementary Information

**Additional file 1.** Interview guide.

## Data Availability

In order to protect the integrity, anonymity and confidentiality of the respondents, data will not be shared.

## Abbreviations

EES: European Economic Area
EU: European Union
IEN: Internationally educated nurses
RN: Registered nurses
