# Supplementary material for: Experiences of nurses educated outside the European Union of a Swedish bridging program and the program’s role in their integration into the nursing profession: a qualitative interview study
Source: BMC Nurs. 2021 Jan 5;20:7. doi: 10.1186/s12912-020-00525-8 (PMC7784301; doi:10.1186/s12912-020-00525-8)
Supplement: Supplementary file 1 — Additional file 1. Interview guide. [file 12912_2020_525_MOESM1_ESM.docx]

**Interview guide**

**Experiences of education and career before arrival in Sweden**

• Please can you tell us about how you came to be a nurse?
• Please can you tell us about your work before the time in Sweden? (Workplaces, tasks, specializations.)
• Please can you tell us about your experiences of working life in Sweden? (E.g. worked as an assistant nurse? In health and care?)
• Please can you tell us about your thoughts on future education at that time? (Goals? Opportunities? Obstacles?)

**Educational experiences from the bridging program**

• How did you find out that the bridging program existed?

• Can you tell us how come you applied for the bridging program?

• Please can you tell us about the application process for the bridging program?
• What expectations did / did you have of the bridging program?
• What are your goals / ideas about what the bridging program will lead to?
• How was the first experience of the bridging program at the university?

• Please tell us about your experiences of the bridging program courses.

• What did you experience as difficult in the bridging program?
• How did you handle difficulties?
• Please can you tell us your experience of the clinical practices?
• Please tell us about similarities and differences compared to your previous professional experience in your home country.
• What were the most important lessons from the practice?
• How did you experience the relationships with colleagues in the clinical practice?

**Expectations of future working and professional life**
• Please tell us about your expectations for the rest of the bridging program.
• Do you think that this education will lead to changes in your life?
• How do you think about your future today? What are your plans / goals? Education? Working life and career opportunities?
• What do you think you should do to achieve your goals?
